# A Critical Review of an Authentic and Transformative Environmental Justice and Health Community — University Partnership

**DOI:** 10.3390/ijerph111212817

**Published:** 2014-12-11

**Authors:** Sacoby Wilson, Dayna Campbell, Laura Dalemarre, Herb Fraser-Rahim, Edith Williams

**Affiliations:** 1 Maryland Institute of Applied Environmental Health, University of Maryland, College Park, MD 20742, USA; E-Mail: swilson2@umd.edu; 2 Department of Health Promotion, Education, and Behavior, University of South Carolina, 921 Assembly Street, Columbia, SC 29208, USA; E-Mail: campbeda@mailbox.sc.edu; 3 Charleston Community Research to Action Board, 2125 Dorchester Rd., North Charleston, SC 29405, USA; E-Mail: herb1373@yahoo.com; 4 Department of Epidemiology and Biostatistics, University of South Carolina, 921 Assembly Street, Columbia, SC 29208, USA; E-Mail: willi425@mailbox.sc.edu

**Keywords:** community-based participatory research, community-campus partnerships, environmental justice, environmental health disparities, environmental health, collaborative problem-solving model, trust, power, communication

## Abstract

Distressed neighborhoods in North Charleston (SC, USA) are impacted by the cumulative effects of multiple environmental hazards and expansion of the Port of Charleston. The Low Country Alliance for Model Communities (LAMC) built an environmental justice partnership to address local concerns. This case study examines the process of building and sustaining a successful transformative and authentic community-university partnership. We apply the framework established by Community-Campus Partnerships for Health (CCPH), focusing on four of the nine principles of *Good Practice of Community Campus Partnerships*.

## 1. Introduction

For the past two decades, Charleston (SC, USA) has ranked as the number two U.S. destination for tourism because of its award-winning restaurants, historic district, beachside resorts and growing economy [1]. However, in the city of North Charleston, low-income, Black communities are experiencing a different type of growth [2]. The expansion of the Port of Charleston and the differential burden of other industries and hazards including Toxic Release Inventory (TRI) facilities, underground storage tanks, brownfields, and Superfund sites have created environmental injustice [3,4,5]. It is well documented that there is an association between socioeconomic patterns of inequity and environmental exposures in the United States. This is no different in North Charleston [6].

The cumulative impacts from multiple environmental hazards as well as the impending expansion of the local port in underserved neighborhoods in North Charleston prompted the development of the Low Country Alliance for Model Communities (LAMC) [3,4,5]. LAMC is a community-based organization consisting of seven economically distressed communities in North Charleston. LAMC works with the University of South Carolina (USC) and the University of Maryland (UMD) to combat known Environmental Justice (EJ) issues which “include the disproportionate burden of environmental hazards on certain populations and communities due to their sociodemographic composition (i.e., race/ethnicity, socioeconomic status) or geography” and related health disparities in the region [3,4,5,7]. The LAMC-USC-UMD Environmental Justice and Health Partnership, also known as the Charleston Pollution Prevention Partnership (CAPs), is focused on capacity-building, grant development, and revitalizing the community [3,4,5,7] using community-based participatory research (CBPR) and the Collaborative Problem-Solving (CPS) Model developed by the United States Environmental Protection Agency (USEPA) [7,8,9,10,11,12,13].

**Figure 1:**
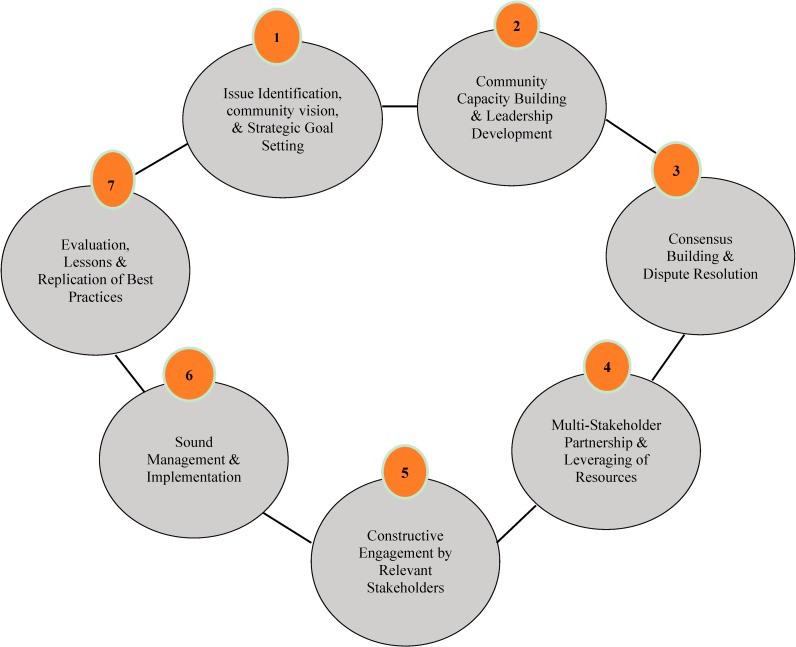
U.S. EPA collaborative problem—solving model.

The Collaborative Problem-Solving (CPS) Model is a community-based approach that stakeholders can use to achieve long-term solutions to local environmental health issues or concerns [8,10,11]. The methodology can be applied to situations that entail collaboration, and can be most useful when dealing with environmental justice issues that are complicated, involve many stakeholders, and where conflicts need to be resolved [14,15,16,17]. Since its introduction, the CPS Model has been used successfully in many situations around the country. There are seven elements in the CPS Model: (1) issue identification, community vision, and strategic goal setting; (2) community capacity-building and leadership development; (3) consensus building and dispute resolution; (4) multi-stakeholder partnerships and leveraging of resource; (5) constructive engagement by relevant stakeholders; (6) sound management and implementation; and (7) evaluation, lessons learned, and replication of best practices. Often viewed as a “tool box” filled with different useful tools, not all the CPS elements are required to be used in every situation. These principles are not intended to be inflexible or to be adopted exact as listed, but instead to provide a starting point or framework for discussion. Our use of the CPS model was modeled after it’s used in Mebane, North Carolina, by the West End Revitalization Association (WERA) [8,10,11]. The WERA CPS partnership was established based on the EPA’s collaborative problem-solving model (see Figure 1) and works within WERA’s Community Owned and managed Research (COMR) framework [10,11]. The WERA CPS partnership consisted of nine working groups that were involved in assessment, management, and corrective action of environmental justice issues specifically the lack of basic amenities in historic, low-income African-American neighborhoods in Mebane, NC, USA [10,11]. The use of the CPS model has encouraged Mebane officials and other government entities to comply with EPA statutes to improve local environmental quality and protect public health.

### Background on Community-Campus Partnerships and CBPR

Community-campus partnerships such as the CAPs partnership can help address issues of concern to the community including environmental injustice and environmental health disparities while providing opportunities for academics to perform applied transdisciplinary research. Research has shown that a strong partnership with communities is a valuable source for teaching, research and practice [14,15]. This interactive process of collaboration is based on common goals that lead to the development of different solutions to community-level problems and concerns. By including the community in research and planning phase, researchers can create programs that have immediate relevance and policy implications [16].

As part of the CAPs partnership, the Community-Based Participatory Research (CBPR) approach was also used. CBPR is defined as “a collaborative approach to research that involves all partners in the research process and identifies the unique strengths that each brings…it begins with a research topic of importance to the community and has the aim of combining knowledge with action and achieving social change” [17]. It has been used successfully in previous community-driven research to address environmental injustice in communities impacted by industrial hog farming in eastern North Carolina [18,19]; traffic and asthma issues in New York [20], landfill issues in underserved communities of color in North Carolina [21,22], and issues with oil refineries in California [23,24].

In fact, research has demonstrated that communities often benefit from partnerships with academic institutions, and that these partnerships have resulted in positive community process outcomes [25] including community empowerment and capacity-building such as the partnership between the West End Revitalization Association (WERA) and faculty and students from the University of North Carolina (UNC) at Chapel Hill [8,9,10,11,12,13,22,26].

In many instances, the partnership can result in the residents organizing to voice their concerns, document experiences, and participate in the recovery process [7,8,9,10,11,12,13,22,26,27] such as residents working with researchers from the USC and Tulane University to address community and worker issues associated with the train derailment and chlorine spill in Graniteville, SC, USA in January 2005 [28].

As an added benefit, the CCP Framework has brought community residents, academic experts and local organizations together for one common goal- to generate culturally appropriate and relevant solutions [25]. It’s been shown that a successful partnership is one characterized by mutuality, supportive leadership, university immersion and asset building, the community [25] and the university both benefit. Alternatively, community-campus partnerships often hold the promise of addressing environmental conditions both on- and off campus, and that those charged with facilitating them need guidance [29], motivation [30,31], the leadership of partners [32,33] and balance of power [34] and trust.

However, CBPR may not always lead to equitable and authentic partnerships because of inequities in power and resources between campus partners and community partners and who is deemed qualified to lead research projects due to institutional cultures associated with research [8,9,10,11,12,13,26]. So, instead of real CBPR, we get community-based research disguised as CBPR. It can be a challenge for community partners to know which campus partner is truly committed to the CBPR principles and which campus partners are exploiting the CBPR principles to maximize process outcomes but not address the social justice and health concerns of partners [8,9,10,11,12,13,26]. Sometimes, community expertise is not as valued as it should be and cultural and community knowledge systems may be marginalized by academics at institutions that supposedly support authentic community-engaged research including CBPR and community-campus partnerships [8,9,10,11,12,13,26].

For example, WERA developed the community-owned and managed research (COMR) framework which focuses on parity in management of the project and equity in funding [8,9,10,11,12,13,26]. COMR’s focus is on the use of scientific data for compliance with environmental regulations and civil rights law [8,9,10,11,12,13,26]. COMR connects community organizing and civic engagement with technical expertise of scientists and lawyers to address environmental injustice [8,9,10,11,12,13,26]. WERA created COMR because their original CBPR partnership with senior researchers at UNC-Chapel Hill was an instance of scientific racism (*i.e.*, use of extractive science to exploit a population of color with known social, economic, and health disparities for academic gain without listening to the community voice or helping to substantively address the problem). WERA’s COMR framework provided a more effective platform for residents to work with partners at the university [8,9,10,11,12,13,26] in developing a shared understanding of problems, generate consensus-based environmental solutions, and advocate for implementation of solutions [29].

Due to WERA’s experience, we believe that as a part of community-campus partnerships, it is important to engage the community by working collaboratively with and through groups of people that share a common geography, special interest, and/or similar situations to address issues affecting their overall well-being [35]. Community engagement is one of the fundamental principles of community-university partnerships that protect the right of the community to engage in making decisions about problems that affect their well-being. This has been shown to be the most effective way to address a problem in a community to involve the members of that community in every aspect that pertains to addressing that problem [35,36,37].

The main purpose of the Principles of Good Community-Campus Partnerships is to help make clear the terms of engagement and expectations between partners [38]. Because partnerships are at various stages of development, the principles provide guidance along the road towards ideal, genuine relationships. The principles are: (1) partners have agreed upon mission, values, goals, and measurable outcomes for the partnership; (2) the relationship between partners is characterized by mutual trust, respect, genuineness, and commitment; (3) the partnership builds upon identified strengths and assets, but also addresses areas that need improvement; (4) the partnership balances power among partners and enables resources among partners to be shared; (5) there is clear, open and accessible communication between partners, making it an ongoing priority to listen to each need, develop a common language, and validate/clarify the meaning of terms; (6) roles, norms, and processes for the partnership are established with the input and agreement of all partners; (7) there is feedback to, among, and from all stakeholders in the partnership, with the goal of continuously improving the partnership and its outcomes; (8) partners share the credit for the partnership's accomplishments; and (9) partnerships take time to develop and evolve over time [38].

The paper reports on the evaluation of a community-academic partnership and its CBPR process in terms of successes and failures, associated with building and sustaining a successful transformative and authentic community-university partnership. It outlines the Partnership’s successes, challenges, and lessons learned over the last three years by reviewing and analyzing the partnership process through the lens of the nine principles of *Good Practice of Community Campus Partnerships*.

## 2. Methodology

Using CCPH’s framework for community-campus partnerships, we identified and addressed principles of *Good Practice of Community Campus Partnerships* [38,39] that best described the CAPs partnership. CCPH’s nine principles of *Good Practice of Community Campus Partnerships* [37] provided a practical framework for evaluating the CAPs structure and process used to address local environmental justice and health issues [40].

We gathered information for this paper through three mechanisms. First, we reviewed all reports and relevant documents that were prepared by each partner from October 2009—September 2012. Second, we asked several members (community leaders and residents, academic partners, and other stakeholders) of our community-campus partnership at a retreat in December 2011 to complete a questionnaire about the partnership. Lastly, key leaders in the organization were interviewed and specifically asked to clarify any gaps in the analysis.

A qualitative data analysis approach was used to review and synthesize all collected data. Using the nine principles of *Good Practice of Community Campus Partnerships* as a guide for themes, we extracted information from each of the reports, documents and questionnaires. This was done be two independent raters and confirmed by a third. To better understand perceptions of the partnership, key leaders were asked to elaborate on each theme and provide a robust commentary on successes and failures. Four of the nine principles emerged as particularly relevant to the characterization of the CAPs partnership and results related to these four principles are reported in the following section.

## 3. Results and Discussion

The following sections provides a detailed outline of the results from the CBPR process in terms of its successes and failures associated with building and sustaining a successful, transformative and authentic community-university partnership. Throughout the process, different principles were applied at different stages. We will discuss the partnership in relation to the following principles; *“Principle 2: The relationship between partners is characterized by mutual trust, respect, genuineness, and commitment”* was used to define and reflect on the LAMC-UMD-USC partnership. Power and funding are discussed as it relates to *“Principle 4: The partnership balances the power among partners and enables resources among partners to be shared and how that fueled power and funding”*. In addition, a communication strategy was used to carry out *Principle 5: There (must be) clear, open and accessible communication between partners, making it an ongoing priority to listen to each need, develop a common language, and validate/clarify the meaning of terms*. During infrastructure building, *Principle 9: Partnerships take time to develop and evolve over time* was used to plan the leadership style, organizational structure, group philosophy, and research approach. We also describe important lessons learned and best practices from the implementation of the CCPH framework for our environmental justice and health community-university partnership.

### 3.1. Trust and Commitment

#### Principle 2: The relationship between partners is characterized by mutual trust, respect, genuineness, and commitment.

Some of the most important components for developing successful and lasting partnerships are trust, mutual respect, sharing of common interests and goals, and the designation of roles and responsibilities. Furthermore, establishing partnerships, particularly between communities and academic institutions, is challenging and is typically fraught with the biases and expectations of both impacted stakeholders and the institutions. These include, but are not limited to, community groups mistrusting the intentions of the institution and researchers viewing their community counterparts as not having the capacity or skills needed for critical engagement and participation in the scientific enterprise. There is some historical basis for these perspectives, but it is paramount to allow positive experiences to counter-balance these perspectives so that the partnership is able to successfully implement the CBPR and CPS principles.

In keeping with the collaborative problem-solving structure of addressing environmental injustice, LAMC established an informal multi-stakeholder partnership with the South Carolina Department of Health and Environmental Control (DHEC) and USC in 2006 [5,7]. Erik Svendsen, state epidemiologist at SCDHEC and assistant professor at the University of South Carolina and John Vena, former chair of the department of epidemiology and biostatistics at USC, were the primary academic partners working with LAMC to provide technical assistance and scientific expertise related to environmental issues of importance [5,7]. They partnered with LAMC to provide research support during the implementation of its mitigation plan agreement.

The critical event in our case study is the expansion of the Port of Charleston. This was the impetus for establishing LAMC, which unified seven North Charleston neighborhoods on one agenda to improve health through revitalization efforts by leveraging mitigation funds. USC was well positioned to engage these communities in empowerment and capacity building efforts to address their environmental health and justice needs. The open forum meetings in the beginning of the process provided all parties the opportunity to share common interests and goals, prioritize needs, and discuss future plans. Three years were spent in discussion between the partners, which is an indicator of the commitment to the collaborative process and the time and effort needed to gain trust and respect [5,6,7].

While the LAMC-USC partnership evolved from the LAMC Mitigation Agreement, implementation efforts of the Mitigation Agreement Committee, and air pollution monitoring efforts of the LAMC-USC-DHEC collaboration, no formal partnership was created by LAMC-USC partnership team members through an agreement such as a Memorandum of Understanding (MOU). In the early stages, the partnership operated on the “bond of Brotherhood” between a few LAMC leaders and the university Principal Investigator (PI) and their mutual commitment to social justice in the African-American community.

In 2009, the team received a four-year, $800,000 grant with Sacoby Wilson as PI and Herb Fraser-Rahim as the community PI from NIH to study: (1) spatial disparities in the distribution of environmental hazards, industrial facilities, and unhealthy land uses in the Charleston region; (2) levels of particulate matter and heavy metals in and across LAMC neighborhoods; and (3) use of CBPR and the CPS model principles to build community capacity to address local EJ and health issues [5,7].

During the first two years, the partnership relied heavily on a few of the “formal” leaders in the community (e.g., neighborhood association presidents, members of the North Charleston city council, city employees, and local business owners). Residents of the various neighborhoods, including those that were not part of the LAMC organization, were not as represented as first thought in the non-leadership layers of LAMC (e.g., the stakeholders who participate in activities, support the organization, and provide the “feet on the ground” performing the “door-to-door” work of the organization).

**Figure 2:**
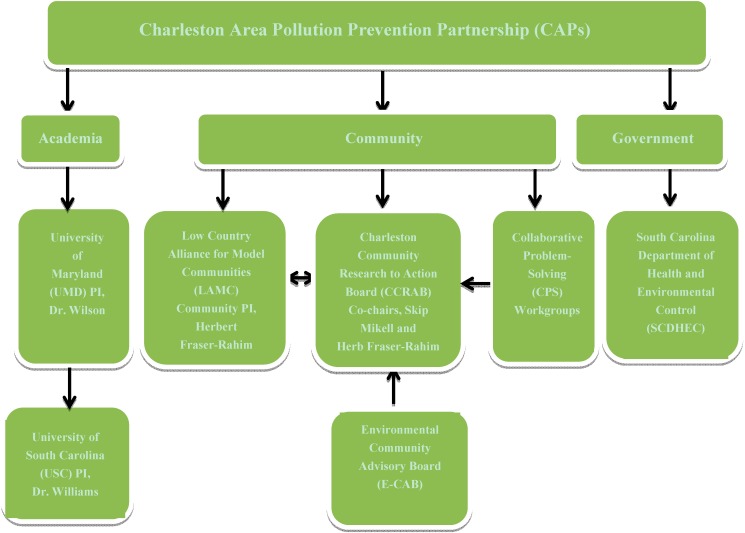
Organizational structure for the community-university partnership.

In reflecting on this principle, academic and community partners learned that personal relationships are the key to an authentic partnership. Individuals in these relationships must show commitment, character, and courage to build a transformative community-campus partnership. By establishing these one-on-one relationships, trust can be built and respect can be earned by both campus and community partners. Unfortunately, to grow the partnership, time and energy must be invested to establish appropriate leadership and management structures and atmosphere of bidirectional engagement and accountability through the use of a Memorandum of Understanding (MOU) or other contractual agreements between community and campus partners that define roles and responsibilities for each partner. Figure 2 provides an organizational structure of the community-university partnership including the primary leadership, academic partners, community partners, and the government partners.

Eventually, a community advisory board was established in 2010 to focus on environmental health concerns related to the NIEHS-funded project for local residents to provide a mechanism for the local community to provide input on the project. This board known as the Environmental Community Advisory Board (E-CAB) had a representative from each community partner who would provide feedback on study goals and the research methodology for each aim, act as conduit for stakeholders in their neighborhood, report back to neighborhood presidents, and provide guidance to the team to make sure we had bidirectional communication between community and academic partners and the collaborative research team adhered to CCPH principles on good partnerships. Unfortunately, there were issues with the E-CAB communication structure which will be explained later.

### 3.2. Power and Funding

Another principle of Good Practice of Community Campus Partnerships [39] that was well-documented in the reports was *Principle 4: The partnership balances the power among partners and enables resources among partners to be shared*.

The issues of power and resources surfaced in the CAPs partnership due largely in part to the competing demands of all partners and mainly because of funding source demands. To address some of these issues, a retreat of stakeholders was held in December 2011 to discuss the restructuring and commitment of a “new” partnership that relied on CPS principles. Members of LAMC, E-CAB, local community, and project staff from USC and UMD attended the event where many decisions about the new direction of the group were made as a group. This meeting was important because it provided a way for academic and community partners to build in more conflict resolution, consensus building, and power sharing in the decision-making process, while also making the partnership more focused on action. In addition this meeting helped the team to begin the process of moving away from a top-down partnership structure driven by a core set of leaders with centralized decision-making authority to one more focused on horizontal, integrated, multi-stakeholder, multi-organizational leadership aligned with the CPS structure.

Additionally, it was also well documented that the departure of the PI from USC resulted in a disruption of grant activities. It also caused some confusion within the partnership, particularly on the part of the community partner. This transition led to some distrust and tension between community leaders and academic team members and also led to tension amongst the LAMC leadership. The shifting of funding to UMD required all of the partners to reassess their roles and responsibilities within the partnership and make decisions about their future involvement in the project. This issue was heightened among community leadership because of past disagreements about the project. This uncertainty led to numerous discussions between the PI and LAMC leadership about the future administration and implementation of activities during years 3 and 4 of the project. Although stakeholders were committed to improving the overall health of North Charleston and the engagement of other communities in the region impacted by environmental injustice and related health disparities, the departure of the PI to the University of Maryland and the addition of this university as a new partner created an imbalance in the power structure. With another university added as a partner, limited financial resources had to be shared across all partners, which led to additional inequities in the funding between university partners and community partners. Additionally, with Maryland providing the primary administrative oversight for the partnership and not having reimbursement processes appropriate for community-university partnerships and community-based participatory research, this made it more difficult for community partners to receive funding they needed to perform their roles and responsibilities on the project.

In order to continue project activities outlined in the grant, the academic partners assumed extra fiscal and task responsibilities. This helped to create an imbalance in power in the partnership because academic partners had more responsibility but less authority in the decision-making process. Conversely, because of the perceived loss of project funding during the grant transfer process, community leadership thought it would have less ownership over the research process, thus less power. The second half of year three was spent re-strengthening old and developing new relationships with community leaders and residents.

The hiring and ongoing training of two community Project Coordinators in 2009 allowed the team to continue balancing power and resources through the exchange of information and skills and has also allowed for a shift of tasks and responsibilities. This proved to be a success in terms of planning and implementing activities related to the project and also providing a “face” for the project that community residents are familiar and comfortable with. The community coordinators work closely with the community PI and academic research team to build community relations, recruit participants, and empower residents while working towards the goal of sustainability.

In reflecting on this principle, community and campus partners agreed that power particularly the sharing of funding was a major issue for the partnership. The inability of the community partner to access funds to cover overhead due to their lack of indirect cost rate (IDC) rate agreement with NIH and the high IDC rate of the campus partner was a source of tension for the partnership. Also, the transition of the PI to another university led to additional funding inequities and tension between partners because of a perceived loss of power and control. Through deliberative dialogue, a plan was established to create a new decision-making structure using the CPS model. Additionally, community project coordinators were hired to ensure that the community concerns were heard and addressed more effectively. The use of the CPS model and the effectiveness of the project coordinators led to mixed results, but addressing structural inequities in power at the partner level and institutional level remains a priority of the community-campus partnership.

### 3.3. Communication

Similar to Principle 2, successful community-university partnerships require clear and open communication based on mutual respect, understanding, and information sharing to ensure their success. *Principle 5: There [must be] clear, open and accessible communication between partners, making it an ongoing priority to listen to each need, develop a common language, and validate/clarify the meaning of terms*, has been the focus of years 3 and 4. The established rules and policies that were negotiated at the beginning of the partnership to avoid misunderstanding had to be revisited and revised continually to ensure success for the final year of the funded project, create solutions, promote sustainability and enhance opportunities for future funding.

CAPs communication relied heavily on the organizational structure developed by LAMC, which is comprised of the neighborhood association presidents and a few outside representatives from each community. Neither LAMC nor the partnership had a communication committee or communication plan. A communication plan outline was developed by the E-CAB, but the outline was not developed into a full plan or implemented fully based on E-CAB recommendations. Information sharing was done solely at the discretion of individual community team members. This included inter-partnership communication and communication with the public. At the start of partnership discussions, this format seemed adequate and was championed by the community partner.

However, it had become clear that the LAMC-USC communication structure and later LAMC-USC-UMD communication structure had a few flaws. Recent miscommunication about changes made to the partnership process and structure following the December 2012 retreat led to some disagreement between partners about the implementation of the CPS structure and workgroups, particularly the use of a stakeholder leadership model instead of the current representative leadership model. March and June 2012 marked the beginning of new dialogue to address the weaknesses in the original partnership, including the lack of a good partnership communication plan. Rules of engagement were established to allow for more equitable decision-making, conflict resolution and better communication with the community and among the stakeholders using the collaborative problem-solving model structure. As part of this structure, the CAPs team created the Charleston Community Research to Action Board (CCRAB).

Currently, CCRAB is evolving into the primary decision-making structure for CAPs with stakeholders and participants signing MOUs stating their roles and responsibilities. In addition, organizational partners signed MOUs. This helps to clarify the relationship between the CCRAB and each individual partner organization, operationalizes CBPR and CPS principles, describes how partners will address problems through conflict resolution and consensus-building, and details how partners will be held accountable for actions related to their ascribed roles and responsibilities.

In reflecting on this principle, partners agreed that effective, consistent, and cyclical communication is a key ingredient of a successful campus-community partnership. Partners learned that poor communication between the community and campus partners and within the community-based organization led to some confusion and tension. This resulted in residents not receiving appropriate updates about the project or inaccurate information. The lack of a well-developed communication plan limited the ability of the E-CAB to be fully engaged in the process. Due to the lack of open and accessible communication particularly from some members of the community partner, a new community-based organization was established to better communicate between the community of concern and campus partner.

### 3.4. Building Infrastructure

Infrastructure Building, which involves the partners creating a working relationship and structure, as well as performing the mission work and creating higher levels of partnership, are key components to CBPR efforts. In accordance with *Principle 9: Partnerships take time to develop and evolve over time*, we recognized that it is essential for a community-university partnership to discuss and agree upon the leadership style, organizational structure, group philosophy, and research approach. This strengthens trust and respect between partners and allows the focus to be solely on the goals and objectives of the project.

Although the E-CAB was established early in the research process, a draft mission and goals statement was developed by the team, but not “owned” and adhered to by all participants. All E-CAB members did not sign an E-CAB agreement as requested nor did they all complete human subjects protection training through CITI as required. The lack of CBPR training for members of the LAMC-USC partnership, including LAMC leaders and CAB members, at the beginning of the partnership and the lack of established group values, mission, and conflict resolution protocol somewhat impeded the ability of the partnership to be more effective at understanding and remedying local environmental health issues.

The overreliance of the partnership on the E-CAB, which consisted of volunteers receiving no compensation for their time, was a central weakness of the Partnership. The E-CAB was established to provide guidance and direct some activities, not necessarily to carry them out. The dependence of the partnership on uncompensated E-CAB volunteers to market project activities and recruit participants has contributed to poor community participation.

The E-CAB and USC decided it was important to create community-level project coordinator positions that would be responsible for carrying out the day to day operations of the project in North Charleston in year 2. The partnership also decided to change the E-CAB to the Charleston Community Research to Action Board (CCRAB) in year 3. This changed the focus of the board from merely a group providing advice but to an action group with decision-making authority. And, these changes have required USC and UMD research staff to be more involved in technical training, and the planning and implementation of community activities. This has been a positive outcome because the community partners benefit more from the technical assistance provided by the academic partners to the CCRAB workgroups while simultaneously providing cross-training to community leadership and stakeholders who participate in CCRAB activities.

Early in the partnership, LAMC lacked capacity and had limited formal policies, procedures, and training to be an effective activism and advocacy organization following CBPR principles. This insight led the academic and community partners into deeper discussions about the need for training and more strategic approaches targeting those communities lacking education and awareness about environmental justice and health issues not related to its original mission.

Over the past year, LAMC has focused on building its organizational capacity by developing policies and procedures for board governance and changing its operational structure so other communities outside of the original seven communities could participate as members of LAMC and become a part of the LAMC leadership. LAMC board members also reflected on the need to adhere to the goal of having proportional representation as outlined in the original mission. The positive changes and internal discussions about organizational capacity-building, development, and growth are due partially to the lessons learned from the Partnership.

The organizational structure was not developed enough for LAMC to be an equal partner with its academic partners in the management and administration of the National Institute Environmental Health Sciences (NIEHS) grant. In addition, it was difficult for the LAMC to provide fiscal oversight of its subcontract because its financial management structure was underdeveloped. The challenges that LAMC has faced during the Partnership have made the organization stronger and better prepared in the future to collaborate with USC and UMD and other outside entities interested in working in community-university partnerships with the organization. It has new policies and procedures in place on collaboration, a better process for fiscal management, and a renewed commitment to addressing environmental health issues beyond issues covered in the Mitigation Plan Agreement.

In reflecting on this principle, the partners agreed that partnerships evolve over time and differences in capacity and resources led to the community partner not being ready to be fully engaged in the CBPR process. Not having an agreed upon vision and mission statement early in the partnership stunted the growth of the partnership particularly because a few community leaders did not buy-in to the process or trust the researchers who were seen as “outsiders”. After LAMC began to do organizational capacity-building and develop a better structure for engaging outside partners, a shared vision for the partnership emerged with individual members of the partnership realizing that CCRAB should be used as the primary interface for community engagement and translation of research to action including more participating of residents in local environmental decision-making.

## 4. Lessons Learned

There were four important lessons learned from our partnership with the community using the CCP framework. One lesson learned is that there is a difference between a formal community leader and a community stakeholder. Both bring value to partnerships and have important roles to play in contributing to development of relationships, building of trust between partners, and ensuring sustainability of efforts to address local environmental health and justice concerns. Partners learned that they have to be open to a new definition of community “leaders” that include those that not only live in the community and participate in representative organizations (e.g., neighborhood associations), but also those individuals who do not hold formal positions. These individuals also desire to see and actively participate in achieving positive social change.

This “new” definition forced us to revisit how we engaged the community, particularly in the areas of participation in partnership activities and communication of activities and successes to different stakeholders in LAMC and sister communities. Developing relationships with non-LAMC and official “leaders” has provided us with greater opportunity to expand and strengthen our partnership.

The second lesson was that the reliance solely on a few people to communicate for the group (e.g., community PI, community project coordinators, and E-CAB members who may have participated inconsistently) proved to be a mistake for the partnership. Understanding and buy-in of goals and objectives for different aspects of the project were not always clearly communicated to all partners and produced confusion. As with any organization, dealing with internal politics of the group can overwhelm and draw attention away from the overall intentions of the project.

On the other hand, there has been a positive response to the development of the CCRAB structure including: (1) more participation of a diverse set of stakeholders from LAMC and non-LAMC communities; (2) a reorganization of the CAPs steering committee; and (3) the creation of a women’s subgroup of female stakeholders interested in environmental health and social justice known as WAEJ (Women Against Environmental (in) Justice). These developments are successful examples of how the community-university partnership has improved communication to more effectively engage impacted stakeholders.

In addition, we learned that building a successful partnership requires examining the organizational structure and capacity of each partner. LAMC originally operated as an “awareness” organization. The goal of awareness-raising activities is to build understanding in the wider community about social justice issues, highlight the work and its importance, and persuade others to become involved as concerned individuals, allies and activists themselves. To be effective in leveraging change in their neighborhoods, the organization must move towards an activism and advocacy organizational structure.

Lastly, we learned that equity in funding between community and university partners should be at the core of all community-university partnerships. Many universities have a wealth of financial resources, staff resources, equipment, and students compared to the limited organizational capacity of community partners. Indirect cost rate (IDC) agreements between universities and federal agencies are agreements that allow for university to receive funds to cover overhead expenses. Unfortunately, community partners usually do not have these agreements with federal agencies and thus are unable to cover their overhead expenses. Additionally, there is a major paperwork burden for community partners to obtain an IDC agreement and due to infrastructure differences, they are put at a financial disadvantage. The inequities in funding between partners can create tension, destabilize relationships, and lead to failure of partnerships attempting to address longstanding environmental justice issues and environmental health disparities in communities of concern. Partners should adhere to COMR principles to ensure that there is equity in funding at all stages of the partnership particularly hiring community leaders to be paid members of the research as community investigators and project coordinators for the duration of the project. Academic partners should work to share their IDCs with community partners or help them complete paperwork so they can have IDC agreements with federal agencies. Academic partners should also provide technical assistance on fundraising to help community partners acquire grants from foundations to build up their short-term and long-term organizational capacity.

## 5. Conclusions

In conclusion, the lessons learned from LAMC’s expansion to address social justice and health issues including and beyond port expansion in North Charleston have been crucial to its growth as an agent of change in the region. As with any organization, it has had its “ups and downs”, and overcoming these challenges has made the partnership between LAMC, USC, and UMD stronger. The open dialogue, power sharing, use of collaborative problem-solving workgroups, and more participation of a diverse set of stakeholders from LAMC and non-LAMC community members has helped strengthen the partnership. Although late in the project, the shift of the E-CAB to a more action-oriented group (*i.e.*, CCRAB) has the potential to bring greater success and lead the community to more sustainability to address local and regional environmental justice and health issues. The growth and expansion of the CAPs partnership has been promoted and supported now by “unofficial” LAMC community leaders (*i.e.*, not elected or members of organizational boards) and non-LAMC stakeholders.

After all evolution is an inevitable part of successful partnerships. Authentic and transformative partnerships at times may have differing levels of tension and distrust between and amongst academic and community partners. Building upon the successes and failures in a continuous cycle can help to create positive social change and sustainability. This process has the potential to transform and empower individuals and the communities in which they live. The negative aspects of this cycle can occur due to: (1) changes in the leadership of academic and community partners; (2) changes in the level of commitment and trust due to natural partner fatigue; (3) power imbalances within and between partners; (4) distribution of and access to financial resources; (5) effectiveness of project management, and (6) internal and external political pressure.

We believe that all community and campus partners should use the CCP Framework to evaluate their partnerships early and often and assess whether or not they are “authentic and equitable” and adhering to the spirit of the CBPR principles. The evaluation could occur in the form of periodic surveys, a quarterly partnership check-in using open-ended questions, or as part of a more formal evaluation annually in a partnership retreat. The principles act as the blueprint for the essential elements of a good community-campus partnership, and without checking the engagement process, structure, activities, and outcomes of the partnership against the principles in a consistent and cyclical manner, the goals and objectives of the partnership may not be achieved and the partnerships could do more harm than good. This is particularly important for academic partners at institutions who have historically not been good neighbors or been drivers of scientific racism or scientific colonialism. Furthermore, partnerships working to address environmental justice issues and health disparities should use the CCP Framework to assess the impact and transformative power of the partnerships at multiple levels- individual, population, community, and policy.
